# Supporting parents following pregnancy loss: a cross-sectional study of telephone peer supporters

**DOI:** 10.1186/s12884-015-0713-y

**Published:** 2015-11-09

**Authors:** Frances M. Boyle, Allyson J. Mutch, Elizabeth A. Barber, Christine Carroll, Julie H. Dean

**Affiliations:** The University of Queensland, School of Public Health, Public Health Building, Herston Road, Herston, QLD 4006 Australia; Sands Australia, Suite 201/901 Whitehorse Road, Box Hill, Vic 3128 Australia

**Keywords:** Perinatal death, Stillbirth, Miscarriage, Bereavement, Peer support, Consumer organisations

## Abstract

**Background:**

The death of a baby before or soon after birth can place an enormous psychological toll on parents. Parent support groups have grown in response to bereaved parents’ unmet needs for support. Peer support is the hallmark of these organisations but little is known about the experiences of volunteers who provide support. This study examines the perceptions and experiences of parent support group volunteers who deliver a 24-h telephone support service for the Australian Stillbirth and Newborn Death Support (Sands) organisation in order to inform the ongoing development and sustainability of effective peer support. This parent-led organisation has delivered support to those affected by miscarriage, stillbirth and newborn death for more than 30 years.

**Methods:**

Twenty-four Parent Supporters completed an online questionnaire. A mix of open- and closed questions asked about aspects of the Parent Supporter role. Quantitative data was summarised using descriptive statistics. Free-text responses to open-ended items were categorised and used to extend and illustrate the quantitative findings.

**Results:**

Our findings reveal a group of highly dedicated and experienced volunteers who had taken 473 calls in the preceding 12 months. Calls were diverse but most were from bereaved mothers seeking ‘to talk with someone who understands’ in the early weeks and months after stillbirth or miscarriage. Most Parent Supporters indicated they felt well-prepared, confident, and satisfied in their role. Challenges include balancing the demands of the role and ongoing training and support.

**Conclusions:**

Peer volunteers contribute to addressing a significant need for support following pregnancy loss. Delivering and sustaining high quality parent-led support depends on volunteer recruitment and retention and this, in turn, requires organisational responses.

**Electronic supplementary material:**

The online version of this article (doi:10.1186/s12884-015-0713-y) contains supplementary material, which is available to authorized users.

## Background

The death of baby is a traumatic and profoundly distressing event. Regardless of when in pregnancy the loss occurs, the grief experienced by parents can be overwhelming with significant and long-lasting repercussions for the wellbeing of families [1–5]. Although many countries have witnessed marked declines in infant and neonatal death rates, pregnancy loss continues to be a reality for large numbers of parents throughout the world. In high-income countries, stillbirth rates have remained steady for two decades with about one of every 200 babies being stillborn [6]. Each year in Australia many thousands of families experience miscarriage, stillbirth or neonatal death. In 2011, 2,562 babies died at or soon after birth: 1,748 babies were stillborn (of at least 20 weeks gestation or with birth weight of at least 400 g) and 814 babies were born alive but died within the first 28 days of life [7]. These figures grossly under-estimate the actual number of pregnancy losses because losses prior to 20 weeks gestation are not recorded. With around 15% of confirmed pregnancies known to end in miscarriage [8] the number of families affected by early pregnancy loss is substantial.

Supportive care is important to psychological recovery following pregnancy loss [9, 10]. Yet, the “invisibility” of these losses, the lack of public recognition and ritual they are accorded, and the social stigma that surrounds them can leave parents without needed support and isolated in their grief [2, 11, 12]. Surveys of parents and health professionals suggest that attitudes, responses and practices in health care settings and the wider community often translate into suboptimal care [11, 13–16]. Lee and Rowlands [17] present a compelling argument that social support deficits are the main determinants of distress after miscarriage.

While pregnancy loss usually occurs in a hospital setting and the actions of maternity staff have important implications for parents’ immediate and longer-term wellbeing, the lived experience of pregnancy loss extends well beyond the hospital stay, which is rarely more than two to three days [16, 18]. Parents may not be ready to comprehend or retain information in the immediate aftermath of their loss and it may not be until after the initial shock subsides, having returned home without a live baby, that the need for emotional support and information becomes paramount. Yet, studies suggest that follow-up care is often inadequate resulting in women feeling abandoned by the health system [3, 19]. An English national survey of 720 women whose infant was stillborn or died soon after birth, identified significant gaps in aftercare and a clear need for the option for continuing post-hospital support [16]. Within Australia, the challenge of providing support is magnified by the vast geographic spread and areas of low population density.

It was in response to unmet support needs that parent-led organisations emerged in the United States in the mid-1970s. Since then the pregnancy and infant loss support movement has expanded rapidly and remains strong [20] with organisations in many countries including the United Kingdom, Ireland, Sweden, Italy and Australia representing the interests of parents and providing bereavement support [21]. More broadly, health consumer and patients’ organisations whose priority is the provision of information, care and support have long been a part of the health care landscape [22]. Kurtz [23] identifies five basic processes that characterise such organisations: giving support; imparting information; conveying a sense of belonging; communication of experiential knowledge and teaching coping methods. Central to these processes, and a defining feature of many pregnancy loss organisations, is the concept of peer support – the provision of support from someone who has experienced the same health problem and has similar characteristics as the proposed recipient [24]. One of the tenets of peer support is reciprocity, where the providers, as well as the recipients, of support stand to benefit through enhanced self-esteem, feelings of altruism, and gaining new perspectives on their own experience [25].

The types and sources of support individual women prefer and have available following pregnancy loss vary and peer support may be particularly important for some [2, 9, 16]. Growing evidence exists for the benefits of peer support [26] but, like other types of support interventions following perinatal loss, rigorous evaluations of the contribution of peer support to psychological outcomes following the death of a baby are scarce [27]. Benefits are widely self-reported by support group participants. These include providing a unique outlet for the sharing of experiences and the telling of stories that help validate and make sense of the loss [2, 19, 20, 28, 29]. Challenges of delivering peer support are also described [24–26]. At the personal level, these include managing high levels of emotional distress and concerns about providing adequate support. Organisational and policy challenges include the recruitment and retention of peer supporters and the provision of training and back-up support in the context of limited resources.

Peer support exists on a continuum: from “lay support” (those to whom people may naturally turn to for support but who have not necessarily experienced the condition) through to “paraprofessional” support (where highly trained supporters may identify with a professional role) [24]. Modes of delivery include face-to-face meetings in group or one-to-one settings, telephone contact and online formats. Aho et al. [30] presented a rare glimpse into the experiences of peer supporters in Finland who joined with a hospital to deliver an intervention to bereaved parents. The study provided valuable insights into personal resources and organisational preconditions that may contribute to the success of such interventions. However, whether findings from a structured service-driven intervention delivered to a defined cohort of parents apply to peer support in more naturalistic settings is uncertain.

Peer support is not intended to replace the expertise of a health professional [24]. In the case of pregnancy loss, high levels of distress are part of the normal grieving process but for a minority of parents specialist care may be warranted [1–5]. Peer support may best be viewed as part of a continuum of care, offering stand-alone support and the ability to augment or facilitate access to formal care as needed. Aho et al. [30] concluded that peer support enabled sustained and intensive support for grieving parents that would have been impossible to organise using professional resources only and surveys of bereaved mothers confirm a lack of post-hospital support services [3, 16].

Although peer support is the hallmark of pregnancy loss organisations, the perspectives of peer supporters are seldom reported. This study aimed to examine the perceptions and experiences of peer support volunteers who operate a 24 h pregnancy and infant loss telephone support service to inform the ongoing development and sustainability of effective peer support.

## Methods

This study formed part of a larger evaluation of support services provided by Sands Australia, a charitable not-for-profit organisation funded by donations and government grants to provide parents, health professionals and the wider community with information, resources and support in relation to the death of a baby at any stage, from conception through to the newborn period. Sands has operated in Australia for more than 30 years and currently has local groups in five Australian states. The organisation is managed by people who have themselves experienced reproductive loss and most are volunteers. Although use of the Internet for bereavement support has expanded within Sands and more widely [31, 32] telephone support remains a core component of support [33].

In 2010, the Australian Government launched an expanded Pregnancy, Birth and Baby Helpline to improve access to birthing and pregnancy related information, including referral to appropriate peer support services for callers seeking assistance following pregnancy loss. As part of this initiative, Sands was funded to provide a national 24-h telephone support service delivered by trained volunteer Parent Supporters. Previously, the organisation had not received specific funding for telephone support and services had operated at the local group level. The new helpline provided a single point of contact for callers anywhere in Australia to reach a Sands Parent Supporter either directly via a dedicated number or by referral from the Pregnancy, Birth and Baby Helpline.

Callers to the support line make direct contact with a Parent Supporter, in their local area where possible. The Parent Supporter provides emotional and informational support and a safe, non-judgmental and caring environment for callers to share their experiences [33]. Sands Australia conducts formal training events, frequently in the form of weekend workshops, for current and prospective Parent Supporters. These focus on developing the knowledge and skills base necessary for effective peer support and include guest presenters with expertise in the field, hands-on skills development and opportunities to explore and reflect on challenges, concerns and strategies. Ongoing individual support is then made available until volunteers are ready for their Parent Supporter role. Parent Supporters are provided with a resource manual and opportunities to debrief as needed with nominated personnel.

### Data collection

A customised questionnaire containing a mix of closed- and open-ended questions was developed with reference to relevant literature and in consultation with Sands Australia representatives to ensure pertinent issues were explored. The questions investigated a range of areas relevant to effective peer support for those affected by pregnancy loss allowing exploration of perceptions and experiences of the Peer Supporter role to inform recommendations for the ongoing development and sustainability of this support service. The 37-item online questionnaire drew on the Peer Volunteer Experience Questionnaire [34] with adaptations to reflect the current context. Items addressed: Training (e.g., “The training provided by Sands has enabled me to be confident in my role as a parent supporter”); Personal effects (e.g., “I find it difficult to balance my role as a Sands Parent Supporter with my other personal/family responsibilities”); Recruitment and Retention (e.g., “I have a clear understanding of what Sands expects of Parent Supporters”); Interactional Characteristics (e.g., “As a Parent Supporter, I feel I can make a difference to other parents who have experienced pregnancy or newborn loss”); and Volunteer role (e.g., “Overall, I find being a Sands Parent Supporter very satisfying). Items were presented as statements, asking participants to respond using a 5-point scale (Always; Most of the time; Some of the time; Rarely; Never). Additional File 1 provides a full copy of the Parent Supporter Survey Questionnaire [see Additional File 1].

Throughout the questionnaire, respondents were invited to make free-text comments in relation to aspects of the Parent Supporter role. Topics included motivations for becoming a Parent Supporter and challenges faced. Examples of open-ended questions were: “What were your main reasons for becoming a Sands Parent Supporter?” and “Regarding the training you have received, what was most useful? How could the training have prepared you better for your role?”

To enhance understanding of the Parent Supporter role, we asked respondents to estimate the number of calls they had received in the last 12 months followed by a series of questions regarding those calls. Each Parent Supporter was then asked to estimate the proportion of those calls (None or very few; Less than half; About half; More than half; All or most) according to a list of call and caller characteristics, including: length of calls (<30 min; 30-60 min; >60 min); identity of the caller (bereaved mother; bereaved father; family member/friend; health professional); and reason for the call (e.g., miscarriage; stillbirth; newborn death). Parent Supporters were also asked what they thought callers were hoping to gain and how callers had heard about the service.

Sociodemographic information about the Parent Supporters (age group; state of residence; length of time in the role and since most recent pregnancy loss; other children; paid workforce participation; and involvement in other activities of the organisation) was collected.

A Sands Australia staff member distributed an information sheet containing a direct link to the online questionnaire by email to all 31 Parent Supporters, followed by two reminder emails. The anonymous survey was hosted and managed by SurveyMonkey, [35] took approximately 20 min to complete and was open to respondents between 25 February and 29 April 2013.

The quantitative data was summarised using descriptive statistics in the form of frequencies as the denominator was less than 100. Parent Supporters provided free-text responses to most open-ended items. Responses, which ranged from a few words to several paragraphs, were read multiple times by two of the researchers who developed an index of codes and themes across the questions. Conceptual consistency of the themes was checked and minimal differences found were resolved through discussion. The themes identified do not necessarily represent the prevalence of particular Parent Supporter responses but rather the extent the theme captures an important aspect of their views and experiences and helps to contextualise the quantitative data. Direct quotes, presented in italics with numbered identifiers (PS1-PS24) to differentiate respondents, are used to illustrate and extend the quantitative results, presenting both themes shared across many Parent Supporters as well as significant experiences identified by only a few.

### Ethics, consent and permissions

Informed consent to participate in the study was obtained by way of a check box at the beginning of the online survey questionnaire. The University of Queensland’s Behavioural and Social Sciences Ethical Review Committee approved the study (#2012001279).

## Results

We present the findings under a series of headings that chart the Parent Supporter journey and integrate quantitative survey data (presented as frequencies) and open-ended responses (presented as illustrative quotes). We examine first the sociodemographic profile of Parent Supporters and their motivations for becoming a Parent Supporter. We then consider the nature of the calls received, their perceptions and experiences of the role, and challenges that relate to effective and sustainable peer support.

### Profile of parent supporters

At the time of the survey, 31 women were registered as Parent Supporters and 24 completed the online survey. The remaining 7 Parent Supporters were not available during the time frame of the survey, usually due to personal circumstances, such as illness or family commitments. Respondents and non-respondents were evenly distributed across the three Australian states in which Sands has had a strong and consistent presence. One Parent Supporter was from Western Australia, reflecting recent efforts to re-vitalise the organisation in that state. As shown in Table 1, most who completed the survey were 40 years or older and had other living children, whose ages spanned the early childhood through to post-high school years. Many combined their voluntary Parent Supporter role with positions in the paid workforce. Most had been a Parent Supporter for at least two years and half for more than 5 years. Consistent with the peer support model, all Parent Supporters had experienced the death of a baby before or soon after birth.

**Table 1:** Characteristics of 24 Sands parent supporters

Parent supporter characteristic	Number
*State of residence*	
South Australia	8
Queensland	8
Victoria	7
Western Australia	1
*Age group*	
20-29 years	1
30-39 years	5
40-49 years	8
50+ yrs	10
*Living children*	
Yes	22
No	2
*Ages of living children* ^a^	
Pre-school	7
Primary school	10
High school	8
Older	10
*Current workforce participation*	
Full-time paid workforce	4
Part-time or casual paid workforce	13
Not currently in paid workforce	7
*Length of time as parent supporter*	
6-12 months	2
1-2 years	2
2-5 years	7
More than 5 years	13

^a^Multiple responses possible

Two-thirds of the Parent Supporters (16 of 24) also reported volunteering for Sands in other ways, with current contributions ranging from 1 to 30 h per week and an average weekly contribution of 7 h. Their volunteering covered a wide range of activities, including: hosting support meetings; presentations to hospital staff; committee membership; assisting with newsletter production; and fundraising. Some had volunteered in ‘*many, many roles over the years*’ (PS1) while others wanted to do more:

*I have small children which can sometimes make it difficult with my time management. I wish that I was available to do so much more for Sands.* (PS18)

### Becoming a parent supporter: *“To share what I had gained”*

The motivation to become a Parent Supporter was driven by personal experience of peer support following pregnancy loss combined with a strong belief in its benefits. All respondents conveyed a deep commitment to ensuring support was available to parents faced with the death of a baby. Some, whose own support had been suboptimal, wanted to ensure better access to support for other bereaved parents:

*When I had my own losses there was nothing like Sands and support available and when I joined I found the support invaluable and wanted to share what I had gained with others in the same situation.* (PS24)

Others wanted to make sure support of the quality they had received continued to be available. Becoming a Parent Supporter also represented a tangible expression of appreciation for support they had received and was a way of “giving back” to the organisation:

*To give back to Sands for all the support they gave us after the loss of our babies. To share my knowledge and skills re grief and loss. To help bereaved parents at their time of greatest need and sadness.* (PS13)

*Wanted to be a support to other parents experiencing grief/loss of a baby. Wanted to give back something back to the organisation that gave me a lifeline in the early days of loss.* (PS14)

A strong emotional connection to their role was evident. For many Parent Supporters, being available to help other bereaved parents was a way of honouring their own babies who had died, giving further meaning to their loss(es):

*To give a sense of purpose to the death of my babies. To be able to give hope to another when it seems that all hope is gone. To walk alongside another through a painful time and feel that my experience of loss is helping someone else survive their loss.* (PS7)

*Being a parent supporter is my way of incorporating my daughter who died into my life.* (PS23)

The Parent Supporter role was a long-term commitment for the women in the study: 20 believed they would still be in the role in 12 months’ time.

### Nature of the calls: *“You can only hope and believe that you have done your role and it has helped”*

The 24 Parent Supporters reported taking a collective total of 473 calls in the last year (range 0 to 100; mean = 20; median = 12). The calls covered a wide spectrum but some categories of calls predominated (Table 2). All estimated that at least half of their calls were from bereaved mothers, and for 14 this was so for all or most of their calls. Calls from bereaved fathers, family members or friends and health professionals represented a much smaller proportion of the total calls. Stillbirth and miscarriage accounted for the largest volume of calls: 15 Parent Supporters said half or more of their calls were in relation to stillbirth and 12 said half or more concerned miscarriage. Although calls following other losses were less common, calls had been taken in relation to a range of pregnancy loss issues including newborn death, medical termination and subsequent pregnancy. The large majority of callers were making contact for the first-time contacts: 22 Parent Supporters reported at least half of their calls were from first-time contacts, including 13 who reported all or most were first-time callers.

**Table 2:** Parent supporters’ reports of the proportion of the calls they had received in the previous 12 months according to selected call and caller characteristics (*n* = 23 due to exclusion of one parent supporter who had not taken any calls in the last 12 months)

	Approximate proportion of calls reported by parent supporters			
Characteristic of callers and calls	All or most	Half or more	Less than half	Very few or none
*Call made by:*				
Bereaved mother	14	9	0	0
Bereaved father	0	3	3	17
Family or friend	0	1	5	17
Health professional^a^	0	2	2	18
*Reason for call:*				
Miscarriage^a^	2	10	9	1
Stillbirth^a^	1	14	4	3
Newborn death^a^	0	4	8	10
Medical termination^a^	0	3	7	12
Ectopic pregnancy^b^	0	0	3	18
Subsequent pregnancy^a^	0	2	2	18
*Time since loss:*				
Less than 1 week^a^	2	4	3	13
2 to 8 weeks	3	10	6	4
2 to 6 months^b^	0	11	5	5
6 to 12 months^a^	0	6	10	6
More than 1 year^b^	0	2	7	12
Longer	0	1	5	16
*Calling for the first time:*				
Yes	13	10	0	0
No (repeat call)	1	3	6	13
*Call answered:*				
Directly^a^	9	8	2	3
In reply to message	1	3	6	13
*Time of call:*				
9am to 5pm^b^	5	11	5	0
5pm to 8pm^c^	1	10	5	3
After 8pm^a^	1	3	8	8
*Length of call*				
Less than 30 minutes^b^	2	5	10	4
30 to 60 minutes^a^	7	11	3	1
More than 60 minutes^b^	0	2	5	14

Not answered by: ^a^1 respondent; ^b^2 respondents; ^c^3 respondents

Callers generally made contact soon after the loss. Thirteen Parent Supporters estimated at least half their callers had made contact in the first 2 to 8 weeks of the loss and 6 estimated that half or more of had done so within the first week. Parent Supporters indicated the typical call was between 30-60 min in length. While most tended to be during business hours, out-of-hours calls were not uncommon.

Parent Supporters identified three main reasons callers contacted the telephone support service: ‘to talk with someone who understands’ (21 of 24 Parent Supporters stated this was a reason for contact “most of the time” or “always”); ‘to gain information’ (19 of 24) and ‘for support in a time of high distress’ (18 of 24). Similarly, they identified two main pathways for callers to the service: referral by hospital staff (doctor, midwife, social worker) (13 of 24 Parent Supporters) and self-referral via the internet (10 of 24).

Calls mainly involved one-off contact, with 14 of 24 Parent Supporters stating that they heard nothing further from the caller after their initial call “most of the time”:

*Sometimes it is frustrating that you only have that call and do not know what happens afterwards, but you can only hope and believe that you have done your role and it has helped.* (PS24)

Most Parent Supporters (22 of 24) became aware that at least some of their callers had further contact with the organisation following the call and 15 reported they had referred at least some callers to other services.

### Being a parent supporter

Parent Supporters expressed a high level of satisfaction with their role (Table 3). They felt valued by the organisation, had a clear understanding of what the organisation expected of them and considered those expectations to be realistic. They felt able to make a difference to other parents faced with pregnancy loss and that their role had helped them to grow personally as individuals and many also gained support for their own experience of pregnancy loss.

**Table 3:** Questionnaire item responses regarding peer supporter experiences: shown as frequencies for the 24 parent supporters

Item	Always or most of the time	Sometimes	Rarely or never
Training provided by Sands has enabled me to be confident in my role	18	6	0
Upon completion of training I clearly understood Sands policies and procedures	19	4	1
Sands values its Parent Supporters	23	1	0
I have a clear understanding of what Sands expects of Parent Supporters	21	2	1
Sands has realistic expectations of Parent Supporters	20	3	1
I have effective strategies to help me "unwind" following a distressing call	18	4	2
I am able to contact someone at Sands for personal support and debriefing after a call if needed	21	2	1
Sands provides opportunities to connect with other Parent Supporters to enhance my role	13	10	1
I feel I can make a difference to other parents who have experienced pregnancy or newborn loss	23	1	0
I feel my role has helped me grow as an individual	22	2	0
As a Parent Supporter, I gain support for my own experience of pregnancy or newborn loss^a^	17	2	4
Overall, I find being a Sands Parent Supporter very satisfying	23	1	0

^a^Not answered by 1 respondent

Most had effective strategies to “unwind” after a distressing call but, notably, six Parent Supporters indicated this was the case only sometimes or rarely. Nevertheless, the majority agreed they were able to contact someone within the organisation for personal support or debriefing if needed.

Responses were slightly more mixed in relation to training. Two-thirds of the Parent Supporters indicated that the training provided had enabled them to be confident in their role and given them a clear understanding of organisational policies and procedures. Fewer respondents (13) felt they had opportunities to connect with other Parent Supporters or members of the organisation to enhance their role.

Free-text comments provided additional insights into views on training, which was regarded in a highly positive light:

*As I am interstate and pretty much on my own without any other peer support workers, being able to attend the training sessions and meeting other Parent Supporters and being able to interact and share everyone’s knowledge and get the personal training was very beneficial.* (PS8)

*I have attended further training sessions and found these helpful to reconfirm my role.* (PS21)

### Challenges related to sustainability

Two main challenges for the sustainability of the service were identified: the multiple demands faced by Parent Supporters and the provision of ongoing training opportunities.

Two-thirds of the Parent Supporters (16 of 24) found it difficult to balance the role of Parent Supporter with other family and personal responsibilities at least “sometimes”, including 5 who said this was the case “most of the time” or “always”. The need for additional Parent Supporters to provide back-up was raised frequently in open-ended responses:

*More trained parent supporters are needed to ease the load* (PS6)

*It would be good if more people were available to share the roster so people could take a break*. (PS22)

Despite the indication that a majority of calls were answered directly (see Table 2), not being able to respond to all calls immediately was a frequently-mentioned concern, with the capacity to produce considerable stress among Parent Supporters:

*It’s difficult sometimes juggling personal life, work, school etc with calls. I hate being at work when a caller has rung and not being able to answer straight away.* (PS15)

*I find that it is quite challenging to incorporate a whole week on roster with my family commitments. I tend to find this week stressful. I know that I have an inability to respond to calls late at night, due to exhaustion and illness. I worry about my inability to respond to calls and find it quite stressful.* (PS19)

For one Parent Supporter, ‘*calls in the middle of the night*’ (PS13) were the most difficult part of her role.

Closely related were Parent Supporter concerns about calls where no message was left. To protect callers’ privacy and to ensure callers communicated about their loss at a time of their choosing, organisational policy dictates that Parent Supporters do not reply to missed calls unless the caller leaves a message explicitly asking them to do so. For one Parent Supporter: ‘*Not being able to make telephone contact after a call where no message has been left*’ (PS16) was the most frustrating aspect of her role.

A strong desire for ongoing skills development and regular contact with other Parent Supporters was a recurrent theme and was considered important for managing issues that might arise and as a supplement to the formal training they had received. The value of ongoing skill development through regular training updates, and opportunities to interact and share practical tips with other Parent Supporters was frequently noted:

*I have drawn upon the training and used it in situations but I have gained mostly through talking and debriefing with other supporters and being able to discuss what to do in situations when they occur.* (PS18)

*The most useful aspect of the training was hearing about specific phone calls taken by more experienced parent supporters, and listening to their tips on combining phone calls with everyday life like kids etc.* (PS2)

Some respondents acknowledged the formidable challenge of providing training across a large and geographically dispersed area with limited resources, pointing to the potential for greater use of electronic technologies, including teleconferences, Webinars, and online training materials to enhance training and networking.

Spontaneous comments from a number of Parent Supporters highlighted a further potential challenge related to the place of peer support in the wider health care arena. It was not uncommon, for example, for callers and referral sources alike to be unaware that their service was provided by volunteers.

*In my experience most of the callers I have spoken with are not aware that* [Parent Supporters] *are bereaved parents. When this is explained to them there is often a "shift", which is often audible in their voice tone and they then appear to generally speak more freely. So I think they gain more from the call than they may be expecting.* (PS9)

*I think at every opportunity we need to make callers aware that they are ringing a volunteer line of people living a normal life, not sitting at a desk waiting for the phone to ring. I have had callers referred from* [another agency] *for example where they are manning the phones 24 h and I’m not sure any of those people have been aware until I told them that I was a normal bereaved parent in my own home.* (PS2)

Taken together, the above challenges reflect the inherent tensions involved in providing a service that combines the features of a volunteer parent-led model of delivery with a strong commitment to highest professional standards and quality assurance.

## Discussion

Our profile of 24 Parent Supporters reveals a group of highly dedicated and experienced volunteers who make significant contributions to support following pregnancy loss. Collectively, they had responded to an estimated 473 calls in the preceding year and, on average, each had also volunteered in other activities to support parents whose baby had died for the equivalent of almost one day per week. The calls taken were diverse – being a 24 h service, there is little predictability in the timing or nature of calls – but most were from bereaved mothers calling for the first time, seeking support, information and understanding in the early weeks and months after stillbirth or miscarriage. This is a time when levels of distress are high [1, 4] and formal follow up care and support is often lacking [3, 13, 16]. Most Parent Supporters indicate they feel well-prepared, confident, and satisfied in their role and that it aligns with their closely held commitment to ensuring support is available for bereaved parents. For many, it also provided a tangible way to honour their own babies who had died.

Alongside the generally high levels of satisfaction reported by Parent Supporters were two main areas of challenge for the sustainability of the service. At the personal level, many Parent Supporters encountered at least some difficulty balancing their role with other commitments. This is not surprising since most combined parenting, paid work and a voluntary role underpinned by a strong commitment and high level of awareness of the need to provide responsive and timely support to bereaved parents. Not being available to take a call or to give the immediate level of support that might be required was a common source of concern. The value of training received and a strong desire for additional and ongoing skills development was a recurring theme. The respondents embraced the concept of peer support and this extended to a peer-based approach to building their skills through mutual learning and connections with other Parent Supporters. Aho et al. [30] reported similarly high levels of demand for training among peer supporters for pregnancy loss.

While the Parent Supporter’s primary role is that of a parent who has experienced the death of a baby, the role also demands the ability to provide support of optimal quality to callers, particularly when contact is usually of a one-off nature. Saflund et al. [36] highlight the importance of ensuring supportive care at various “turning points” following stillbirth while Downe et al. [14] point out that health care providers usually have “only once chance to get it right” by responding with warmth and humanity. The accumulation of positive healing memories, including responses that convey warmth and humanity, contributes to parents’ psychological recovery. Although these studies are focused on clinical care, arguably their findings are equally relevant to support in the wider post-hospital environment.

It is difficult to specify the ideal characteristics of peer supporters [26, 30]. Having the skills and confidence to provide emotional support, recognise the limits of peer support, refer to other services and engage in self-care is essential as is the ability to relate and communicate across a wide range of circumstances. Approaches to training need to be considered carefully for services that are founded on the principles of peer support [24–26]. Training should harness the potential of shared experience to contribute to recovery by providing support that is qualitatively different from that offered by other types of health professional contact. Simultaneously, peer supporters must be equipped with essential skills to respond to difficult and highly emotional content and to balance emotional involvement and empathy [26], especially in the context of their own loss experiences.

The extent to which similarity between peer supporters and those they support influences the quality of support is uncertain [30]. All of the Peer Supporters and most of their callers in our study were women. Pregnancy loss support groups traditionally are women-dominated [20] and the support needs of men have received minimal attention. Men were only occasional users of the telephone support service and, as others suggest [30], the question of whether peer support might be better tailored to meet men’s needs requires further attention. Addressing the needs of people from culturally and linguistically diverse backgrounds and other minority groups is a consideration in relation to access, including peer supporters recruited from different backgrounds. In relation to pregnancy loss, potentially value-laden reasons for contacting support services such as therapeutic termination of pregnancy raise the possibility that personal or religious views may influence the support provided. In practice, the potential for matching on personal characteristics is likely to be limited due to the range of pregnancy loss experiences, the finite number of Parent Supporters available at any one time and the caller-driven nature of the 24 h telephone support line.

The insights gained and challenges identified are relevant to broader organisational level issues relating to the recruitment, support, retention and training of adequate numbers of volunteers. Ensuring back-up support by enlisting more volunteers in a climate where volunteering may be decreasing and where voluntary organisations face significant financial pressures is a challenge shared by many non-profit organisations [22]. In Australia, the geographical distance between Parent Supporters and their organisations magnifies some of these challenges. Online support options continue to advance and provide new and extended avenues of support [31, 32]. However, the fundamental qualities of peer support are equally relevant across different modalities whether telephone, face-to-face or internet.

### Strengths and limitations

Our study provides a detailed and rarely available picture of the experiences of volunteer peer supporters who provide frontline telephone support for bereaved parents. The evaluation was conducted independently of Sands but, consistent with the principles of community-based research, the questionnaire was developed collaboratively and findings were presented to organisational representatives for member checking, increasing confidence that our account is accurate and comprehensive. However, caution must be taken in generalising the findings they pertain to a particular setting and group of volunteers. Most had been in the role for some years and since our study is confined to current Parent Supporters, the views of those who may have chosen not to continue in the role are not included. The short timeframe available to complete the evaluation meant it was not feasible to document calls prospectively, nor was it feasible to obtain retrospective records from Parent Supporters. All data relied on recall and self-report, offering a subjective account of Parent Supporter experiences.

## Conclusion

Peer support services provide a unique opportunity to gain ready access to information and support from a parent who has also experienced the death of a baby. Our findings suggest that peer support delivered by parent-based organisations addresses a significant need following pregnancy loss, particularly for recently bereaved mothers who represented the majority of those seeking support.

Delivering and sustaining high quality parent-led support depends on volunteer recruitment and retention. By documenting the experiences of Parent Supporters, our study highlights issues that require careful consideration at organisational levels. Parent Supporters expressed high levels of satisfaction and a strong commitment to their role. However, they also identified several key challenges centring mainly on training, opportunities for ongoing mutual learning and back-up support.

Further research is needed to develop an evidence-base to guide policy and practice in this area. In-depth interviews with Parent Supporters about the benefits and challenges of their role could illuminate areas for training and support, with consideration to Parent Supporters’ experiences of processing their own grief and loss alongside their role of supporting others and their perceptions and responses to different forms and circumstances of pregnancy loss. Most importantly, further information is needed on the perceptions, experiences and outcomes of peer support from the perspective of support recipients as well as those who have experienced pregnancy loss but have not accessed support services. Such information is critical to developing socially and culturally sensitive support that is responsive to the wide diversity of support needs.

## Additional file

Additional file 1:
**Parent Supporter Survey Questionnaire.** (PDF 248 kb)

## References

[1] Boyle FM, Vance JC, Najman JM, Thearle MJ (1996). The mental health impact of stillbirth, neonatal death or SIDS: prevalence and patterns of distress among mothers. Soc Sci Med.

[2] Cacciatore J (2013). Psychological effects of stillbirth. Semin Fetal Neonatal Med.

[3] Geller PA, Kerns D, Klier CM (2004). Anxiety following miscarriage and the subsequent pregnancy: A review of the literature and future directions. J Psychosom Res.

[4] Neugebauer R, Ritsher J (2005). Depression and grief following early pregnancy loss. Int J Childbirth Educ.

[5] Rådestad I (2001). Stillbirth: care and long-term psychological effects. British J Midwifery.

[6] Flenady V, Middleton P, Smith GC, Duke W, Erwich JJ, Khong TY (2011). Stillbirths: the way forward in high-income countries. Lancet.

[7] Australian Bureau of Statistics (2013). Causes of Death, Australia, 2011 (cat. no. 3303.0). ABS: Canberra.

[8] Regan L, Rai R (2000). Epidemiology and the medical causes of miscarriage. Clin Obstet Gynaecol.

[9] Hutti MH (2005). Social and professional support needs of families after perinatal loss. J Obstetric Gynecol Neonatal Nurs.

[10] Cacciatore J, Schnebly S, Froen JF (2009). The effects of social support on maternal anxiety and depression after stillbirth. Health Soc Care Community.

[11] Frøen JF, Cacciatore J, McClure EM, Kuti O, Jokhio AH, Islam M (2011). Stillbirths: why they matter. Lancet.

[12] Rowlands IJ, Lee C (2010). ‘The silence was deafening’: Social and health service support after miscarriage. J Reprod Infant Psychol.

[13] Cacciatore J (2010). The unique experiences of women and their families after the death of a baby. Soc Work Health Care.

[14] Downe S, Schmidt E, Kingdon C, Heazell AEP (2013). Bereaved parents’ experience of stillbirth in UK hospitals: a qualitative interview study. BMJ Open.

[15] Gold KJ (2007). Navigating care after a baby dies: a systematic review of parent experiences with health providers. J Perinatol.

[16] Redshaw M, Rowe R, Henderson J (2014). Listening to Parents after stillbirth or the death of their baby after birth.

[17] Lee C, Rowlands IJ (2014). When mixed methods produce mixed results: Integrating disparate findings about miscarriage and women’s wellbeing. Br J Health Psychol.

[18] Gold KJ, Sen A, Xu X (2013). Hospital costs associated with stillbirth delivery. Matern Child Health J.

[19] Kelley MC, Trinidad SB (2012). Silent loss and the clinical encounter: Parents’ and physicians’ experiences of stillbirth–a qualitative analysis. BMC Pregnancy Childbirth.

[20] Layne L (2006). Pregnancy and infant loss support: A new, feminist, American, patient movement?. Soc Sci Med.

[21] Heazell AEP, Leisher S, Cregan M, Flenady V, Frøen JF, Gravensteen IK (2013). Sharing experiences to improve bereavement support and clinical care after stillbirth: report of the 7th annual meeting of the international stillbirth alliance. Acta Obstet Gynecol Scand.

[22] Baggott R, Jones K. (2014). The voluntary sector and health policy: The role of national level health consumer and patients' organisations in the UK. Soc Sci Med. doi:10.1016/j.socscimed.2014.07.016 10.1016/j.socscimed.2014.07.016 25085720

[23] Kurtz LF (1997). Self-help and support groups: A handbook for practitioners.

[24] Dennis CL (2003). Peer support within a health care context: a concept analysis. Int J Nurs Stud.

[25] Pistrang N, Jay Z, Gessler S, Barker C (2013). Telephone peer support for women with gynaecological cancer: benefits and challenges for supporters. Psycho-Oncology.

[26] Fisher EB, Coufal MM, Parada H, Robinette JB, Tang PY, Urlaub DM (2014). Peer support in health care and prevention: Cultural, organizational, and dissemination issues. Annu Rev Public Health.

[27] Koopmans L, Wilson T, Cacciatore J, Flenady V (2013). Support for mothers, fathers and families after perinatal death. Cochrane Database Syst Rev.

[28] McCreight BS. (2007). Narratives of pregnancy loss: The role of self-help groups in supporting parents. Medical Sociology Online, 2, 3–16

[29] Umphrey LR, Cacciatore J (2011). Coping with the ultimate deprivation: Narrative themes in a parental bereavement support group. OMEGA-J Death Dying.

[30] Aho AL, Åstedt-Kurki P, Kaunonen M (2014). Peer supporters’ experiences of a bereavement follow-up intervention for grieving parents. OMEGA-J Death Dying.

[31] Gold KJ, Boggs ME, Mugisha E, Palladino CL (2012). Internet message boards for pregnancy loss: Who’s on-line and why?. Women's Health Issues.

[32] van der Houwen K, Stroebe M, Schut H, Stroeb W, van den Bout J (2010). Online mutual support in bereavement: An empirical examination. Comput Hum Behav.

[33] Sands – miscarriage, stillbirth & newborn death support. http://www.sands.org.au/. Accessed 27 October 2015.

[34] Dennis CL (2003). The effect of peer support on postpartum depression: a pilot randomized controlled trial. Canadian Journal of Psychiatry/Revue Canadienne De Psychiatrie.

[35] Surveymonkey.com, (2015). SurveyMonkey: Free online survey software & questionnaire tool. http://surveymonkey.com. Accessed 27 October 2015.

[36] Saflund K, Sjogren B, Wredling R (2004). The role of caregivers after a stillbirth: views and experiences of parents. Birth.

