# Supplementary material for: Supporting parents following pregnancy loss: a cross-sectional study of telephone peer supporters
Source: BMC Pregnancy Childbirth. 2015 Nov 9;15:291. doi: 10.1186/s12884-015-0713-y (PMC4640395; doi:10.1186/s12884-015-0713-y)
Supplement: Additional file 1: — Parent Supporter Survey Questionnaire. (PDF 248 kb) [file 12884_2015_713_MOESM1_ESM.pdf]

Sands Parent Supporters Survey

About this survey

Thank you for your interest in this survey. The purpose of the survey is to learn about the important role of Sands Parent Supporters. We want to find out what is working well and what could work better.

Participation in this survey is completely voluntary. Please read the Information Sheet that was sent to you by email for more details about the study. If you would like another copy of the Information Sheet, please let us know and we will send one to you.

If you decide to participate in this research survey, you may choose to withdraw from the research at any time- just close your web browser. Filling out the online survey will take about 30-40 minutes. Your responses will be confidential and viewed only by the research team based at The University of Queensland. We do not collect identifying information such as your name, email address or IP address. All data is stored in a password protected electronic format.

Please note this survey is not a test – there are no right or wrong answers. Answers that reflect your own views, ideas and experiences will help us to identify ways to make the Sands telephone support service the best it can be. There will be both tick-box type questions and questions where you can enter as much or as little detail as you choose. We'll be asking about your role and experiences as a Parent Supporter as well as information about the calls you've responded to. You might find it helpful to have your case notes handy to jog your memory.

You may leave the survey and come back (using the same computer) as many times as you like until you select 'done' on the final page. This feature means that only one person can complete the survey on a shared office computer. Alternatively you can complete the survey from at home or the library or you may print the survey, hand write responses and return it to us by post, email or fax.

If you have any questions or need any assistance please contact either:
Liz Barber (e.barber@uq.edu.au)
Fran Boyle (f.boyle@sph.uq.edu.au) tel: 07 3346 4681 or mob: 0402 099 556

Thank you for taking the time to complete this survey. We value your responses.

*1. Clicking on the “agree” button below indicates that:

You have read the above information
You voluntarily agree to participate

If you do not wish to participate in the research study, please decline participation by clicking on the “disagree” button.

- ◯ Agree
- ◯ Disagree

Sands Parent Supporters Survey

Practical aspects of being a Parent Supporter

These questions are about the practical aspects of your role as a Sands Parent Supporter and the training and support you have in that role. Your answers will help us to understand what areas are working well and whether there are any areas that could be improved.

2. Training

	Never	Rarely	Sometimes	Most of the time	Always
The training provided by Sands has enabled me to be confident in my role as a parent supporter	◯	◯	◯	◯	◯
Upon completion of the training I clearly understood Sands policies and procedures	◯	◯	◯	◯	◯

3. Please add any further comments about the training you have received. What was most useful? How could the training have prepared you better for your role as a parent supporter?

4. About the roster

	Never	Rarely	Sometimes	Most of the time	Always
I am rostered on for the right number of shifts	◯	◯	◯	◯	◯
The roster system works well	◯	◯	◯	◯	◯
During my rostered shift, I am able to respond to calls immediately	◯	◯	◯	◯	◯

5. Please make any suggestions as to how the roster system might work better.

6. Policies and processes

	Never	Rarely	Sometimes	Most of the time	Always
The shift timeframe/duration of time on 'calls' is about right	◯	◯	◯	◯	◯
The roster system enables me to respond to messages in a timely way	◯	◯	◯	◯	◯
The new Returning Missed Calls/Voicemail policy works well	◯	◯	◯	◯	◯
The 'call sheet' is easy to use to record caller information	◯	◯	◯	◯	◯
I understand why it is important to collect some information about callers	◯	◯	◯	◯	◯
I find it easy to adhere to Sands' privacy and confidentiality policies	◯	◯	◯	◯	◯
There is always someone at Sands that I can contact with questions, concerns and reflections arising from calls	◯	◯	◯	◯	◯

Sands Parent Supporters Survey

7. Parent Supporter role

	Never	Rarely	Sometimes	Most of the time	Always
I have a clear understanding of what Sands expects of Parent Supporters	◯	◯	◯	◯	◯
Sands has realistic expectations of Parent Supporters	◯	◯	◯	◯	◯
Sands values its Parents Supporters	◯	◯	◯	◯	◯
I am able to contact someone at Sands for personal support and to debrief about a call when I need to	◯	◯	◯	◯	◯
Sands provides opportunities to enhance my capacity as a Parent Supporter through connections with other Parent Supporters/Sands staff	◯	◯	◯	◯	◯

8. Please make any suggestions as to how Sands might better assist you and/or other Parent Supporters in delivering telephone support.

9. We are interested in your views on whether the Parent Supporter role has changed over time. In the past few years, have you observed any changes in your role as a Parent Supporter (eg number of shifts rostered for, amount of training/professional development, networking, referral pathways, paperwork) or the nature of the calls you receive (eg length of call, time of day, characteristics of callers, concerns, level of distress)?

- ◯ Yes
- ◯ No
- ◯ I became a parent supporter recently

If yes, please describe

Sands Parent Supporters Survey

Your role as a Parent Supporter

The following questions are about you and how you feel about your role as a Sands Parent Supporter. Learning about Parent Supporters is important to understanding how best to support and maintain this vital part of the Sands community.

10. Your experience as a Parent Supporter

	Never	Rarely	Sometimes	Most of the time	Always
I feel that my role as a Parent Supporter has helped me grow as an individual	◯	◯	◯	◯	◯
I feel that, by volunteering as a Parent Supporter, I gain support in relation to my own experience of pregnancy or newborn loss	◯	◯	◯	◯	◯
As a Parent Supporter, I feel I can make a difference to other parents who have experienced pregnancy or newborn loss	◯	◯	◯	◯	◯
I find it difficult to balance my role as a Sands Parent Supporter with my other personal/family responsibilities	◯	◯	◯	◯	◯
I have effective strategies that help me 'unwind' following a distressing call	◯	◯	◯	◯	◯
Overall, I find being a Sands Parent Supporter very satisfying	◯	◯	◯	◯	◯
I feel sure I will be a Sands Parent Supporter in 12 months time	◯	◯	◯	◯	◯

11. What were your main reasons for becoming a Sands Parent Supporter?

12. What do you like most about being a Parent Supporter?

13. What do you dislike or find frustrating about your role as a Parent Supporter?

14. Can you foresee any reasons why you might stop volunteering with Sands?

About the callers

The following questions are to help us to gain an overall understanding of the callers you have supported. We are interested to know how callers use the Sands telephone support service and to gain a better understanding of their varied needs. We don't need precise answers- an estimate is fine.

15. In your experience, what do callers most often hope to gain by contacting the Sands telephone support service?

	Never	Rarely	Sometimes	Most of the time	Always
To talk with someone who understands	◯	◯	◯	◯	◯
Support in a time of high distress	◯	◯	◯	◯	◯
To defuse a crisis	◯	◯	◯	◯	◯
To develop coping strategies	◯	◯	◯	◯	◯
To gain information and/or advice	◯	◯	◯	◯	◯
To find out what Sands offers	◯	◯	◯	◯	◯
To gain information about other services (e.g., counselling)	◯	◯	◯	◯	◯

Other (please describe)

16. In your experience, how do callers tend to hear about the Sands telephone parent support service?

	Never	Rarely	Sometimes	Most of the time	Always
Referred by hospital (e.g., doctor, midwife, social worker)	○	○	○	○	○
Referred by GP	○	○	○	○	○
Referred by other community services	○	○	○	○	○
Family or friends	○	○	○	○	○
Internet	○	○	○	○	○

Other (please describe)

____

17. In your experience, how often do the following happen after a call?

	Never	Rarely	Sometimes	Most of the time	Always	Don't know
The caller contacts you again	○	○	○	○	○	○
The caller has further contact with Sands	○	○	○	○	○	○
You contact the caller again	○	○	○	○	○	○
You hear nothing further of the caller	○	○	○	○	○	○
You refer the caller to another service	○	○	○	○	○	○
The caller becomes (or considers becoming) a member of Sands	○	○	○	○	○	○

Other (please describe)

____

About the calls

The following questions are to help us to gain an understanding of the varied nature of calls you respond to. Again, we're looking for estimates, not precise numbers.

18. About how many calls have you taken as a Sands Parent Supporter over the past year?

Estimated number of calls

19. If you have been in the role for less than one year, please state the length of time you have been a Parent Supporter (please estimate the number of calls you have taken in Question 18 above).

Number of months

Thinking about all the calls you have taken over the past year, for the questions below please check the response that most closely applies to those calls.

20. Length of calls

	None or very few	Less than half	About half	More than half	All or most
Calls less than 30 minutes in length	○	○	○	○	○
Calls 30 minutes to 60 minutes in length	○	○	○	○	○
Calls longer than 60 minutes	○	○	○	○	○

21. Timing of calls

	None or very few	Less than half	About half	More than half	All or most
Calls before 9am	○	○	○	○	○
Calls between 9am and 5pm	○	○	○	○	○
Calls between 5pm and 8pm	○	○	○	○	○
Calls after 8pm	○	○	○	○	○

22. Response to calls

	None or very few	Less than half	About half	More than half	All or most
Answer initial calls directly	◯	◯	◯	◯	◯
Return calls following message	◯	◯	◯	◯	◯
First time callers	◯	◯	◯	◯	◯
Repeat callers	◯	◯	◯	◯	◯

23. About the callers

	None	A few	Less than half	About half	More than half	All or most
Calls made by bereaved mothers	◯	◯	◯	◯	◯	◯
Calls made by bereaved fathers	◯	◯	◯	◯	◯	◯
Calls made by friend or family member on behalf of bereaved parent	◯	◯	◯	◯	◯	◯
Calls from health professionals on behalf of bereaved parent	◯	◯	◯	◯	◯	◯
Calls from health professionals seeking general advice	◯	◯	◯	◯	◯	◯

24. In relation to their loss

	None or very few	Less than half	About half	More than half	All or most
Calls in relation to miscarriage	◯	◯	◯	◯	◯
Calls in relation to newborn death	◯	◯	◯	◯	◯
Calls in relation to stillbirth	◯	◯	◯	◯	◯
Calls in relation to medical/genetic termination	◯	◯	◯	◯	◯
Calls in relation to ectopic pregnancy	◯	◯	◯	◯	◯
Calls in relation to a subsequent pregnancy	◯	◯	◯	◯	◯

25. Time since loss

	None or very few	Less than half	About half	More than half	All or most
Calls within one week of loss	◯	◯	◯	◯	◯
Calls in the first 6-8 weeks of loss	◯	◯	◯	◯	◯
Calls 3-6 months after loss	◯	◯	◯	◯	◯
Calls 6-12 months after loss	◯	◯	◯	◯	◯
Calls more than 1 year after loss	◯	◯	◯	◯	◯
Calls some years after loss	◯	◯	◯	◯	◯

About you

These next questions will help us to gain a profile of Sands Parent Supporters across Australia. Remember, all your responses are completely confidential.

26. I am in my

- ◯ 20s
- ◯ 30s
- ◯ 40s
- ◯ 50s+

27. My postcode is

Postcode ______

28. I have been a Parent Supporter for

- ◯ Less than 6 months
- ◯ 6-12 months
- ◯ 1-2 years
- ◯ 2-5 years
- ◯ More than 5 years

29. How long was it between your most recent pregnancy loss and commencing as a Sands Parent Supporter?

Years ______

Months ______

30. Do you have other living children?

◯ Yes

◯ No

31. Select the age category/ies of your living child/ren

- [ ] pre-school
- [ ] primary school
- [ ] high school
- [ ] older

32. I am currently in the:

- ◯ full-time paid workforce
- ◯ part-time or casual paid workforce
- ◯ not currently in paid workforce

33. Apart from your role as a Parent Supporter, do you volunteer in other ways for Sands? If yes, please list any other ways you volunteer for Sands (e.g., hosting coffee mornings; member of committee; assisting with newsletter)

- ◯ Yes
- ◯ No

If yes, please describe

34. On average, how many hours per week do you work as a Sands volunteer?

Average hours per week

35. On average, how many hours per month are you rostered on as a Parent Supporter?

Hours per month

Your ideal Parent Support service

In this section we're keen to hear about your ideas or 'vision' for Sands Parent Support Service. This is an opportunity to share any recommendations, concepts, strategies or technologies that you feel would make this service even better.

36. In an ideal world (with no limits in terms of human, financial or other resources), what would Sands Parent Support service look like? (For example, you may consider opportunities to use social networking, technology, ways to improve links with services and service providers, nature of volunteering/paid work, training and professional development, others.)

37. Have we left something out? Please add any further comments.

Finished!

Thank you for your time and for sharing your thoughts and experiences. We encourage you to contact us if you think of other details that would contribute to this evaluation. Please contact: Liz Barber (e.barber@uq.edu.au) or Fran Boyle (f.boyle@sph.uq.edu.au) tel: 07 3346 4681. Thanks also for your dedication to Sands and the vital support you provide to our community.
